# Fabricating sub-nanometer materials through cluster assembly

**DOI:** 10.1039/d2sc03813g

**Published:** 2022-09-26

**Authors:** Qingda Liu, Xun Wang

**Affiliations:** Department of Chemistry, Key Lab of Organic Optoelectronics and Molecular Engineering, Tsinghua University Beijing 100084 China wangxun@mail.tsinghua.edu.cn

## Abstract

The self-assembly of clusters provides a feasible approach for the bottom-up fabrication of functional materials with tailored properties. Sub-nanometer cluster assembly with a well-defined construction presents a precisely controllable structure and extraordinary properties, which provides an ideal model for the investigation of structures and properties at the molecular level. Non-covalent interactions between clusters may dominate the assembly behavior, appearing as tunable structures different from their nano-counterparts. Interactions between clusters and their superatom orbitals can significantly influence the electronic structures, because of which exceptional properties may emerge. In this paper, recent progress on cluster-based assemblies is introduced, including sub-nanometer building blocks of noble metal and polyoxometalate (POM) clusters. The structures, formation mechanism and properties of these cluster assemblies are discussed from experimental and theoretical aspects. This perspective aims to provide a new insight into the design and manufacture of sub-nanometer materials based on clusters.

## Abstract 2

This perspective discusses the synthesis and precise control of sub-nanometric cluster assemblies at the molecular level, as well as the exceptional properties derived from interactions between clusters.

## Graphical abstract

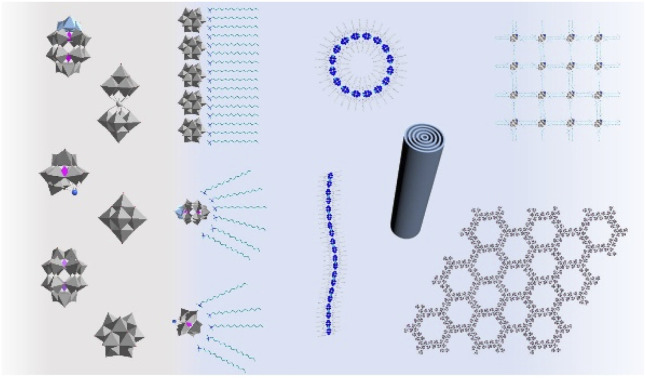

## 1. Introduction

Clusters, as a wide class of atomic precision entities residing between atoms and nanocrystals, feature various structures and diverse functions in different fields.^1–9^ In addition to the design and growth of new types of clusters, the self-assembly of clusters offers an alternative approach to the functionalization of nanoclusters with collective and customized properties.^10–13^ Clusters with strict monodispersity and a rugged construction are ideal building blocks, while their assembly may not favor the formation of closely packed phases, due to the specific geometry and surface properties of clusters.^14–17^ As sub-nanometer particles, covalent or non-covalent interactions between clusters can dominate their solution behaviors, because of which hierarchical assemblies in multiple dimensions have been realized following different synthetic strategies.^18–22^ In addition, interactions between cluster building blocks can significantly affect their electronic structures and properties, leading to the enhanced properties of cluster-based assemblies compared to individual building blocks.^23–25^

Polyoxometalate (POM) and noble metal clusters are sub-nanometer metal oxide and metal nanoparticles, respectively, with atomic precision structures and geometries.^26,27^ As the most representative cluster species, the synthesis and assembly of POM and noble metal clusters have been comprehensively studied in the past few decades.^28–31^ These two types of clusters show similar inspiring properties as the desired building blocks for self-assembly: (1) the negatively charged clusters of 1–2 nm that can act as macromolecules in solution, showing variable assembly behaviors that can be precisely controlled by weak interactions. (2) The solubility of clusters can be tuned by changing cation counter ions, which is applicable in many solvent systems. (3) The satisfactory stability in water and organic solvents allows the employment of multiple methods for their assembly and characterization. Compared with noble metal clusters, metal substitution of POMs results in overturned surface property changes in charge and coordinate properties, which can be enlarged with the encapsulation of surface ligands. With the use of anisotropic POM building blocks and two-phase synthesis, cluster assemblies can be confined to the sub-nanometer scale. These single-cluster assemblies display well-defined constructions with tunable structures and exceptional functions, providing a rational model for the in-depth understanding of the structure–property relationship at the molecular level. In addition, the use of other types of clusters as building blocks has also been reported. Due to the lack of functional groups, fullerene-based assemblies are commonly achieved by the covalent modification of fullerene on the surface.^32,33^ Meanwhile, host–guest interactions usually serve as the main driving forces to build a cucurbituril-based complex, contributed by their unique geometry.^34,35^ The solvophobic and host–guest interaction-driven assembly in these systems usually results in large-sized nanomaterials with limited morphology tunability.

In this perspective, we will review recent advances in the self-assembly of noble metal and POM clusters, and discuss the critical factors during the synthesis. The following sections involve the development of cluster materials from a crystal phase to sub-nanometer assemblies, and the interesting properties derive from the unique construction of sub-nanometer cluster assemblies. The assembly behavior of a single cluster and the surroundings is analyzed by theoretical methods, which offers an instructive insight into the design and synthesis of cluster assemblies from the molecular level. Advanced functions may arise from non-covalent interactions and super-atom orbital interactions between clusters and surface ligands. Finally, the future prospects for sub-nanometer cluster assembly are outlined.

## 2. From crystals to nanostructures

### 2.1. Noble metal clusters

**Fig. 1:**
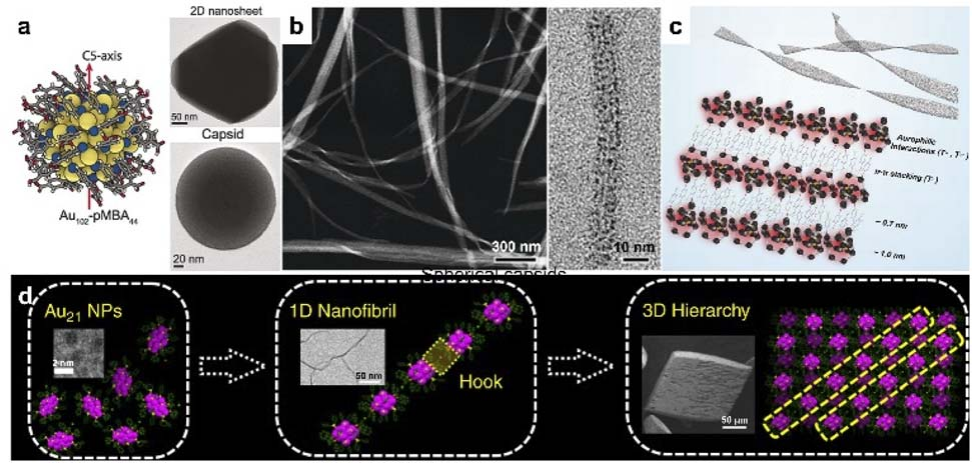
. Noble cluster assemblies. (a) Colloidal crystallization of Au_102_-*p*MBA_44_ clusters into 2D nanosheets and spherical capsids. Reproduced from ref. ^37^. (b) HRTEM images of nanoribbons. (c) Schematic illustration of self-assembly of Au_25_(*p*-MBA)_18_^−^ into nanoribbons. Reproduced from ref. ^38^. (d) The hierarchical fibrous (1D to 3D) assembly of Au_21_ clusters. Reproduced from ref. ^39^.

Noble metal clusters can be considered as ultrafine nanoparticles with strict monodispersity. For the clusters with high symmetry, the typical assembly in colloidal systems may favor the formation of closely packed phases. Therefore, the rational use of surface ligands may be essential to break symmetry and construct low-dimensional nanostructures with desired properties. For example, the encapsulation of *p*MBA (*para*-mercaptobenzoic acid) allows hydrogen bonding between clusters, which benefits the formation of colloidal superstructures and provides an available site for the modulation of cluster interactions. Lee and Xie reported Ag_44_-*p*MBA_30_^4−^ supracrystals with tailored shapes by modifying the binding environment of clusters.^36^ The rhombohedral supracrystals (*D*_3d_ symmetry) would change into octahedral ones (*O*_h_ symmetry) by replacing the counterion of the *p*-MBA ligand from H^+^ to Cs^+^, which eliminated the directional hydrogen bonds in supracrystals. And the increase in Cs^+^ concentration could also shape the supracrystals into concave octahedra. Later, Ikkala *et al.* revealed the assembly of Au_102_-*p*MBA_44_ into hexagonally packed 2D faceted colloidal crystals in methanol, with a monolayer thickness of 2.7 nm.^37^ Closed spherical capsids of 200 nm were also obtained under controlled solvent conditions, driven by the nonspherical arrangement of hydrogen bonding that promotes planar packing. Through the tuning of pH in the aqueous phase, isolated Au_25_(*p*-MBA)_18_^−^ clusters were able to transform into nanoribbons of 10–50 nm in width and several micrometers in length (Fig. 1b).^38^ The assembly was initiated by surface-motif reconstruction of Au_25_(*p*-MBA)_18_^−^ from short SR–[Au^I^–SR]_2_ units to long SR–[Au^I^–SR]_*x*_ (*x* > 2), which directed the anisotropic organization into 1D nanowires. π–π stacking further promotes the aggregation of individual nanowires into nanoribbons (Fig. 1c).

Oriented cluster assembly can also be applied with other ligands and solvent systems. Jin and co-workers constructed the hierarchical fibrous (1D to 3D) assembly of Au_21_ in a pentane/dichloromethane system, by tailoring the surface ligands and associated counterions (Fig. 1d).^39^ Au_21_ clusters were initially assembled into 1D nanofibrils with site-specific surface hooks, including π–π, anion–π, and aryl C–H⋯Cl interactions. The fibrous assembly would evolve further into 3D crystals through the slow diffusion of pentane into the dichloromethane dispersion. In another study, 1-dodecanethiol-protected Au_15_ clusters were used as building blocks for self-assembly into mono-, few- and multi-layered sheets in colloidal solution.^40^ The dipolar interaction induced the anisotropic assembly into a 1D-oriented assembly. The asymmetric distribution of surface ligands leads to asymmetric vdW attraction, resulting in the formation of 2D nanosheets with tunable length, aspect ratio and thickness. Moreover, Xie's group realized a series of spherical micelle assemblies by using a unimolecular star-like β-CD-*g*-P4VP-*b*-PS diblock copolymer as the ligand.^41^ The size of assemblies could be well controlled by the molecular weight of block polymers. Clusters including Au, Ag, Cu, Pt and AuAg alloy with a certain size (<2 nm) were used as building blocks, indicating the generality of this process. The cation ligand of CTAB can also direct the directional assembly of metal clusters. Zeng *et al.* reported 3D hierarchical assemblies, which were highly dependent on metal precursors and CTAB concentration.^42^ One-dimensional Au cluster nanorods could restructure into hollow vesicles with additional CTAB, and Pt cluster giant vesicles would evolve into a dandelion-like spherical morphology at a lower CTAB concentration. Rhombic/hexagonal Pd cluster assemblies were generated from vesicle coalescence. In 2015, Lee and Xie prepared amphiphilic cluster building blocks Au_25_(MHA)_18_@*x*CTA *via* a phase transfer approach,^43^ which was realized by an ion-paring reaction between hydrophobic cations (CTA^+^) and the anionic surface groups (COO^−^) of hydrophilic clusters. The amphiphilic clusters could assemble into a stacked bilayer structure with regular interlayer packing at the air–liquid interface.

The above studies involve noble metal clusters with different symmetries, surface ligands with different functional groups and solvent systems with different polarities. And the driving forces of cluster self-assembly include hydrogen bonds, electrostatic interactions, van de Waal forces and solvophobic interactions. Restricted by the metal atom covered surface, noble metal clusters are not able to connect directly by weak interactions. Atom doping in noble metal clusters mainly affects the electronic structures, but little the surface properties. All these assemblies are constructed *via* weak bonds between surface ligands, where clusters are separated by double-layered surface ligands. Due to the non-directional encapsulation of surface ligands, the arrangement of surface ligands on clusters is not fixed, and the intrinsic asymmetry of the cluster shall be weakened, which is adverse to the oriented assembly at the sub-nanometer scale. For these reasons, vesicles, layered structures or supracrystals are commonly observed, and one-dimensional sub-nanometer assemblies are difficult to obtain in metal cluster-based assemblies.

### 2.2. POM clusters

Unlike noble metal clusters, hydrophilic POM clusters with an oxygen-rich surface can bond with metals, amino/quaternary ammonia groups, carboxyl groups and other molecules through covalent or non-covalent interactions. Thus, a wide class of POM-based assemblies are fabricated through rational control over the multiple interactions. Bare giant POM clusters (with alkali cations or NH_4_^+^) can slowly aggregate into blackberry structures in aqueous solution, driving by van de Waals forces.^44,45^ The coating of organic ligands with long hydrophobic alkyl chains on POM clusters results in amphiphilic building blocks that are compatible with organic systems. The use of synthetic methods, POM cluster types and surface ligands with different symmetries can significantly influence the morphologies of POM assemblies. By covalent grafting of a hydrophobic alkyl chain (C_15_H_31_) on MnMo_6_O_24_ clusters *via* amide bonds, the hybrid POM cluster can form vesicles in acetonitrile/water solvents.^30^ Similar vesicular structures can be built from other Dawson and Lindqvist-type clusters with the modification of different organic molecules.^46,47^

**Fig. 2:**
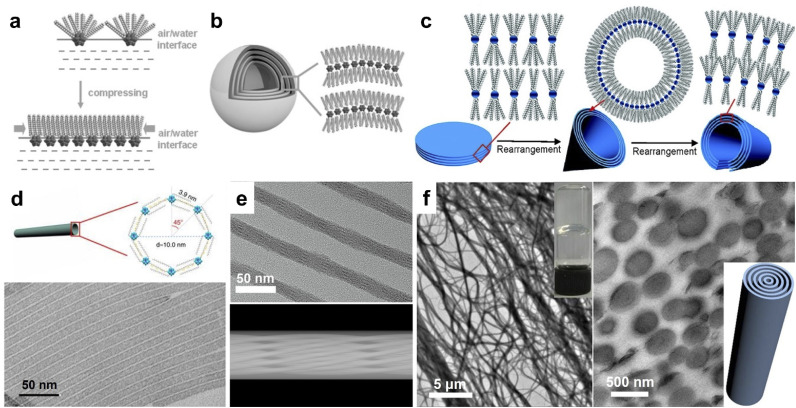
Amphiphilic POM assemblies. (a) Rearrangement of surface ligands on POM clusters at the air–water interface. (b) Onionlike spherical assembly of (DODA)_4_SiW_12_O_40_. Reproduced from ref. ^50^. (c) Reversible self-assembly of (DODA)_3_PW_12_O_40_/(DODA)_3_PMo_12_O_40_ between nanodisks, nanocones, and nanotubes. Reproduced from ref. ^52^. (d) [PW_12_O_40_]^3−^ single-walled nanotubes. Reproduced from ref. ^54^. (e) HRTEM image and 3D model of (TTA)_6_P_2_W_18_O_62_ helical nanotubes. Reproduced from ref. ^55^. (f) TEM image and cross section of (CTA)_3_(TBA)_3_P_2_W_18_O_62_ supramolecular gel. Reproduced from ref. ^56^.

Non-covalent encapsulation of POM clusters with cation surface ligands has proved to be an efficient way to construct amphiphilic assemblies in different dimensions. DODA (dioctadyl dimethyl ammonium bromide) with double alkyl chains is commonly used to prepare 2D and 3D POM assemblies. The assembly of amphiphilic clusters at the liquid–water interface leads to the compression of surface ligand cations on the POM cluster (Fig. 2a), which favors the formation of layered structures. Keggin type [XW_12_O_40_]^*n*−^ (X = H, B, P, Si, Co) clusters can self-assemble into monolayer films at the air–water interface using the Langmuir–Blodgett technique.^48^ Liu *et al.* reported the assembly of DODA and Keplerate clusters into a highly ordered honeycomb at the air–water interface.^49^ The assembly of (DODA)_8_[Eu(H_2_O)_2_SiW_11_O_39_] clusters appears as vesicles in chloroform, but micrometer sized honeycomb structures on solid supports.^21^ Wu *et al.* synthesized the onion-like spheres of (DODA)_4_SiW_12_O_40_ assemblies by controlling the solvent polarity (Fig. 2b).^50^ By the use of triple-chained ammonium ions comprising 2-(methoxyethoxy)ethyl, Kimizuka *et al.* prepared giant 2D nanosheets with Keggin-type clusters [PM_12_O_40_]^3−^ (M = Mo, W).^51^ The nanosheets show plane rectangular shapes containing long (1.0–3.5 μm) and short (0.5–2.5 μm) sides, with a thickness of 15–30 nm. The giant size and dissolution of reduced POM clusters also allow the photoetching and photodeposition of Ag on the predetermined area of nanosheets. Under solvothermal conditions, our group developed the reversible assembly of (DODA)_3_PW_12_O_40_ and (DODA)_3_PMo_12_O_40_ (Fig. 2c), which can transform between nanodisks, nanocones, and nanotubes in chloroform/butanol with different volume ratios.^52^ In other mixed solvents, these building blocks can form rose-like (tetrahydrofuran/*n*-butanol), snow-like (acetone/*n*-butanol), and ice-ball (butanone/*n*-butanol) assemblies with the same layer spacing,^53^ which is due to the rearrangement of DODA on POM clusters in different polarity solvents.

The use of single alkyl chain ligands, including those of amines and quaternary ammoniums, benefits the formation of 1D superstructures. With the mixture of Keggin type [PW_12_O_40_]^3−^ clusters and oleic acid/oleylamine, single-walled nanotubes could be prepared under solvothermal conditions (Fig. 2d).^54^ Based on the same synthetic method, the assembly of OTAB/CTAB/TTAB/DTAB (octadecyl-, cetyl-, tetradecyl-, and dodecyl-trimethylammonium bromide) and [PW_12_O_40_]^3−^ clusters was confirmed to be semi-tubes and nanowire bundles with lamellar constructions.^57^ Interestingly, the ionic combination of Dawson type [P_2_W_18_O_62_]^6−^ clusters and TTA^+^ appeared as helical microporous nanorods (Fig. 2e).^55^ There exist a set of parallel fringes in a helical nanorod constructed by the rotation of interwoven channels along the axial direction. By introducing double surface ligands, we have also fabricated (CTA)_3_(TBA)_3_P_2_W_18_O_62_ and (CTA)_3_(TBA)_3_P_2_Mo_18_O_62_ supramolecular gels under mild conditions (Fig. 2f).^56^ The Dawson POM-based nanowires with a circular cross section are crosslinked at the nanometer scale, which strikingly increases the dispersion viscosity into a transparent supramolecular gel.

Among these assemblies, the solvent system (synthetic method) and surface ligand symmetry are decisive factors in the formation of nanosized assemblies. The amphiphilic building blocks tend to form monolayered structures in high-polarity solvents, but spheres or aggregated assemblies in the presence of low-polarity solvent, which is attributed to the specific arrangement of surface cations on POM clusters. Due to the strong interactions between alkyl chains, double chain ligands prefer the parallel alignment on POM clusters (Fig. 2a–c). The reduced curvature of POM layers leads to the generation of layered films and large spherical assemblies. Meanwhile, single chain ligands with discrete distribution around clusters allow the formation of ultrasmall micelles, because of which the assemblies can be shaped in the radical direction with a confined size.

## 3. Sub-nanometer POM assembly

Interactions between building blocks are difficult to identify in large-sized cluster assemblies, which is detrimental to structural tunability and in-depth investigation of the structure–property relationship. In this section, we will discuss the construction of single-cluster assemblies with well-defined molecular models and tunable structures and properties at the molecular level. In the following studies, two-phase synthesis is applied, where POM precursors dissolved in water would mix with a chloroform phase containing CTAB/TBAB ligands under vigorous stirring at room temperature. During the reaction, POMs together with possible small molecules would transfer into the chloroform phase as amphiphilic clusters, which could further self-assemble into sophisticated assemblies under controlled conditions.

### 3.1. Solution behaviors during phase transformation

Before introducing the structures and properties, we first pay attention to the interactions between POM clusters, surface ligands and solvents. In colloidal systems, electrostatic interactions, coordination bonds, van der Waals force and hydrogen bonds usually serve as the main driving force to dominate self-assembly. Due to the lack of connective sites, interactions between POM clusters with high symmetry (such as intact Keggin and Dawson type) are non-directional. Their self-assembly into nanosized materials is directed by surface ligands and solvent properties, as summarized in Section 2.2. In order to construct cluster assemblies with sub-nanometer size, or in other words, single cluster thickness, the oriented assembly is essential. Therefore, metal-substituted POM clusters are selected as anisotropic building blocks, and a series of sub-nanometer cluster assemblies have been successfully prepared by water–chloroform two-phase synthesis.

**Fig. 3:**
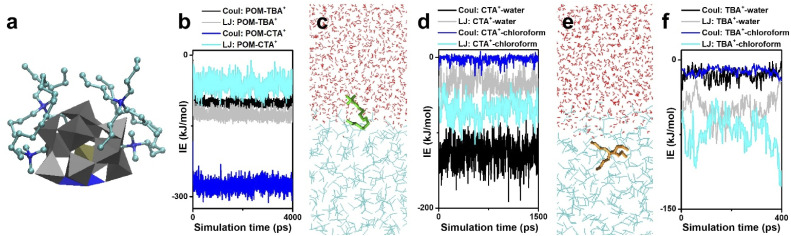
Interactions between POM clusters, surface ligands and solvents. (a and b) MD simulations (a) and interaction energies (b) of POM and surface ligands (CTA^+^ and TBA^+^). (c and d) CTA^+^ at a water–chloroform interface (c) and the relevant interaction energies (d). (e and f) TBA^+^ at a water–chloroform interface (e) and the relevant interaction energies (f). Reproduced from ref. ^58^.

In this part, mono-metal substituted Keggin type clusters [M^III^PW_11_O_39_]^4−^ are taken as the building block, and the solution behavior of POM clusters during phase transformation is studied by molecular dynamics (MD) simulations. In interaction energy analysis, Coul stands for the electrostatic interactions and LJ stands for the Lennard-Jones potential, which contains the non-electrostatic interactions such as Van de Waals forces, solvophobic interactions, dipole–dipole interactions, *etc.* The encapsulation of surface ligands (2CTA^+^ and 2TBA^+^) on POM clusters is first investigated in vacuum (Fig. 3a). The electrostatic interactions (IE) between POM and surface ligands are much stronger than the LJ potential (Fig. 3b) and serve as the major driving force to dominate the assembly process. Due to the shape and symmetry of the surface ligand, the electrostatic attraction of POM–CTA^+^ is stronger than that of POM–TBA^+^, while the steric-hindrance effect of TBA^+^ is more significant than that of CTA^+^. Driven by the electrostatic attraction, CTA^+^ and TBA^+^ can spontaneously attach to the surface oxygen of the POM cluster, and the surface oxygen atoms far from the M atom are shielded by the long alkyl chains. However, due to the repulsion between positive charges, the M atom in addition to adjacent oxygen atoms is exposed, which becomes a specific coordinative site for the inter-cluster connection into linear configurations.

Surface ligands with different shapes exhibit different behaviors in the two-phase system. CTA^+^ prefers to stay at the water–chloroform interface, where the cation head is located in the water and the alkyl tail faces the chloroform (Fig. 3c). The Coul of CTA^+^–water and the LJ potential of CTA^+^–chloroform are the main forces to keep this balance (Fig. 3d). On the other hand, TBA^+^ tends to go deep into the chloroform, which is due to the weaker electrostatic interactions and stronger LJ potentials between TBA^+^ and chloroform (Fig. 3e and f).

**Fig. 4:**
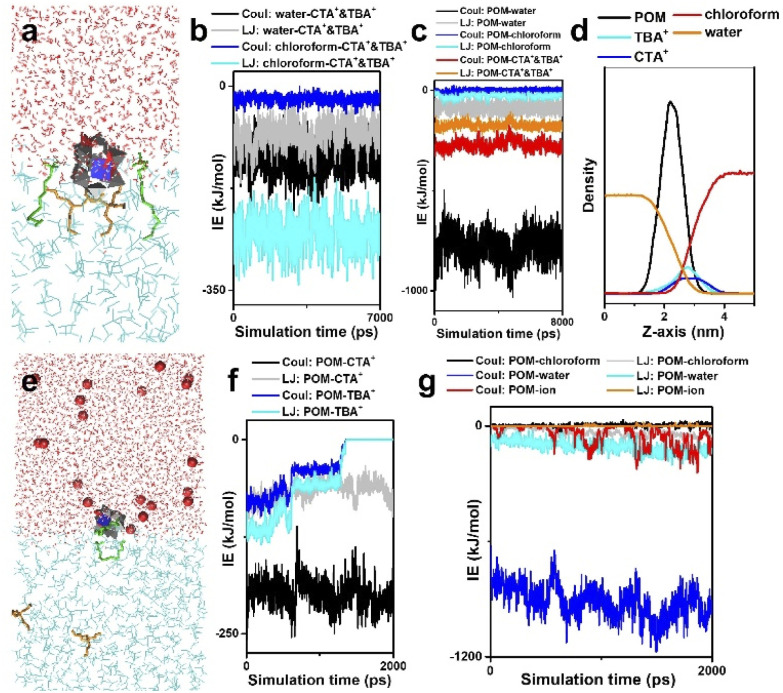
Solution behavior of a surface ligand encapsulated single POM cluster. (a) MD simulation on POM–surface ligands at a water–chloroform interface. (b) Interaction energies between surface ligands and solvents. (c) Interaction energies between POM and surroundings. (d) Density distributions of POM and surface ligands at the water–chloroform interface along the *z*-axis. (e) Solution behavior of POM–surface ligands in the presence of excess metal ions. (f) Interaction energies between POM and surface ligands. (g) Interaction energies between POM and surroundings. Color code: red ball, excess metal ions; red, water; cyan, chloroform; green, CTA^+^; orange, TBA^+^. Reproduced from ref. ^58^.

We further move to the solution behavior of the surface ligand encapsulated single POM cluster, where one POM cluster together with two CTA^+^ and two TBA^+^ is placed near the water–chloroform interface. During the phase transfer, the POM cluster is captured at the water–chloroform interface driven by surface ligands (Fig. 4a). Interaction energies between solvents and building blocks (Fig. 4b and c) demonstrate that (1) there are strong electrostatic interactions between POM and water, for which the POM unit prefers heading to the water phase; (2) surface ligands prefer staying in the chloroform during the assembly; (3) contributed mainly by the LJ potential of surface ligand–chloroform and the electrostatic attraction of POM–surface ligands, CTA^+^ and TBA^+^ serve as binders to pull POM clusters to the water–chloroform interface. According to the density distributions of the above components (Fig. 4d), the range of the water–chloroform interface (overlapping of red and orange lines) is close to the size of a single cluster, which may efficiently confine the assembly of the POM cluster into the sub-nanometer scale.

Note that part of the POM cluster is exposed in the water phase, and the addition of small molecules and cations may influence their self-assembly through interactions at the molecular level. Next, we focus on the solution behaviors of POM–surface ligands, where excessive metal ions are introduced into the water phase (Fig. 4e). Due to electrostatic repulsion, competitive adsorption between surface ligands and metal ions causes a significant configuration change of POM–surface ligand units. POM–CTA^+^ can still be stabilized at the interface, while TBA^+^ is pushed into the chloroform. The separation of TBA^+^ results in the exposure of surface oxygen in POM clusters, providing more connective sites for potential linkage between building blocks.

On the basis of theoretical methods, the two-phase approach has proved to be a feasible way to construct single-cluster assemblies under mild conditions. With the encapsulation of surface ligands, the uneven charge distribution in metal-substituted POM clusters is transformed into asymmetry in geometry and surface properties, where the uncovered metal site with positive charge benefits the oriented assembly in one-dimension. The assembly at the water–chloroform interface with a sub-nanometer size further confines the cluster assembly into a single cluster range. The use of two types of quaternary ammonium with different properties allows precise control over the solution behavior of clusters through interactions at the molecular level.

### 3.2. Tunable structures at the molecular level

**Fig. 5:**
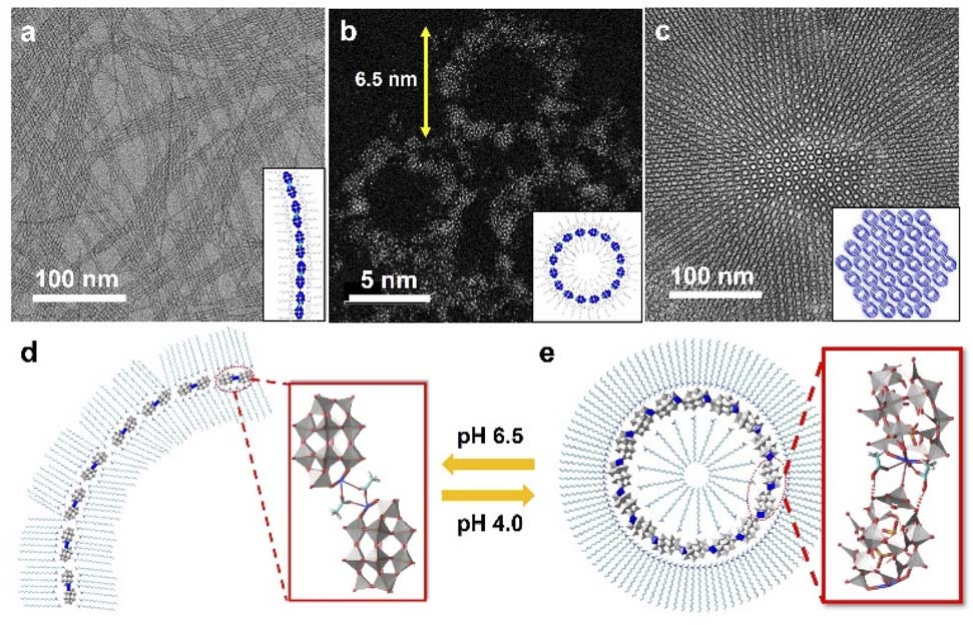
POM single-cluster assemblies with tunable structures. (a) TEM image of POM single-cluster nanowires. (b) HRAC (high-resolution aberration-corrected)-TEM image of POM single-cluster nanorings. (c) TEM image of 3D superstructure constructed from nanorings. (d) Molecular model of a single-cluster nanowire. (e) Molecular model of a single-cluster nanoring. Reproduced from ref. ^59^.

Following this strategy, our group developed a series of single-cluster nanowires, nanorings and 3D superstructures with well-defined structures and molecular models.^59^ The assembly behavior of POM clusters can be precisely controlled by step-wise tuning of interactions at the molecular level. A mono-metal substituted Dawson type cluster [P_2_W_17_MO_61_]^7−^ was used as the building block, and acetic acid/acetate was introduced as the molecular liner. In a neutral pH (6.5) water phase, POM clusters could self-assemble into single-cluster nanowires with an ultrahigh aspect ratio (Fig. 5a). In the nanowire morphology, POM clusters are connected *via* acetate in a head-to-head configuration to form dimers, and these dimers are linearly aligned into nanowires through van der Waals forces and electrostatic interactions by surface ligands (Fig. 5d). When the pH is decreased to 4.0, single-cluster nanorings were obtained, with a uniform diameter of 6.5 nm (Fig. 5b). In weak acid solution, POM dimers are connected *via* molecular linkers of acetic acid, which prefers a head-to-tail configuration with a specific angle of 22° between adjacent clusters (Fig. 5e). The nanorings could therefore be constructed in a closed topology with 16 clusters. Single-cluster nanorings could further assemble into 3D superstructures in a few days, with size up to 1 μm (Fig. 5c). In addition, 15 types of metal-substituted POM clusters can be used to manufacture similar structures, indicating the general feasibility of this assembly process.

**Fig. 6:**
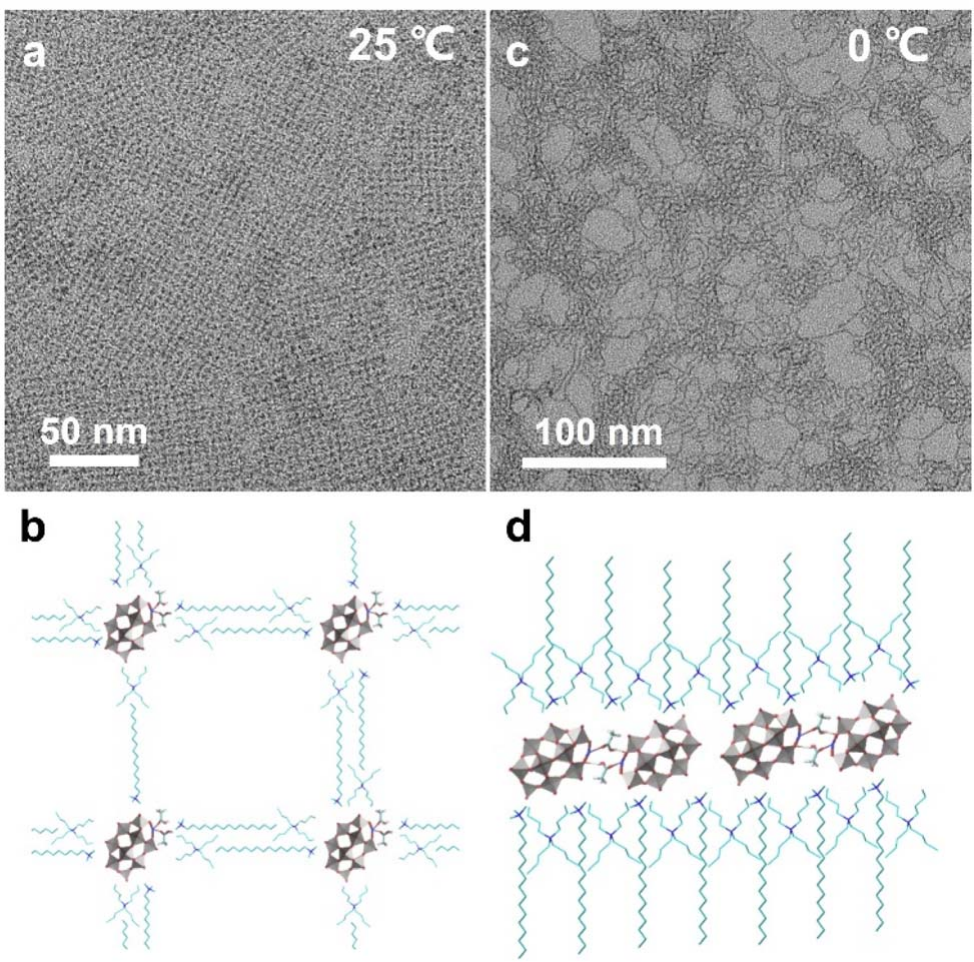
Temperature-responsive POM assemblies. (a and b) TEM image and molecular model of 2D superlattice structures at 25 °C. (c and d) TEM image and molecular model of single-cluster nanowires at 0 °C. Reproduced from ref. ^60^.

Weak interactions between POM clusters and their surroundings can have a non-negligible impact on the solution behaviors and properties of single clusters. Thus, the single cluster assemblies may show higher sensitivity to the external environment and variable and reversible assembly behaviors that are different from their nano-counterparts. In recent work, our group reported temperature-responsive single cluster assemblies driven by hydrogen bonds using the same synthetic method.^60^ The morphology transformation can be applied reversibly at near room temperature. Notably, no temperature-responsive ligands are involved and the stimuli-responsive process is driven by hydrogen bonds between cluster building blocks. Here, a Mn-substituted Dawson type cluster [P_2_W_17_Mn^III^O_61_]^7−^ is used and acetic acid serves as the molecular linker. The single-cluster assembly can spontaneously arrange into 2D superlattice on supports at 25 °C (Fig. 6a), and POM clusters exist as monomers with the coordination of acetic acid (Fig. 6b). When the temperature is decreased to 0 °C, the POM assembly transforms into single-cluster nanowires (Fig. 6c), where POM clusters form dimers in a head-to-head configuration through the hydrogen bonds of acetic acid linkers (Fig. 6d). The nanowires show a higher aspect ratio and straighter geometric construction at lower temperature (−10 °C), due to the weaker thermal motion of clusters.

### 3.3. 2D clusterphenes

**Fig. 7:**
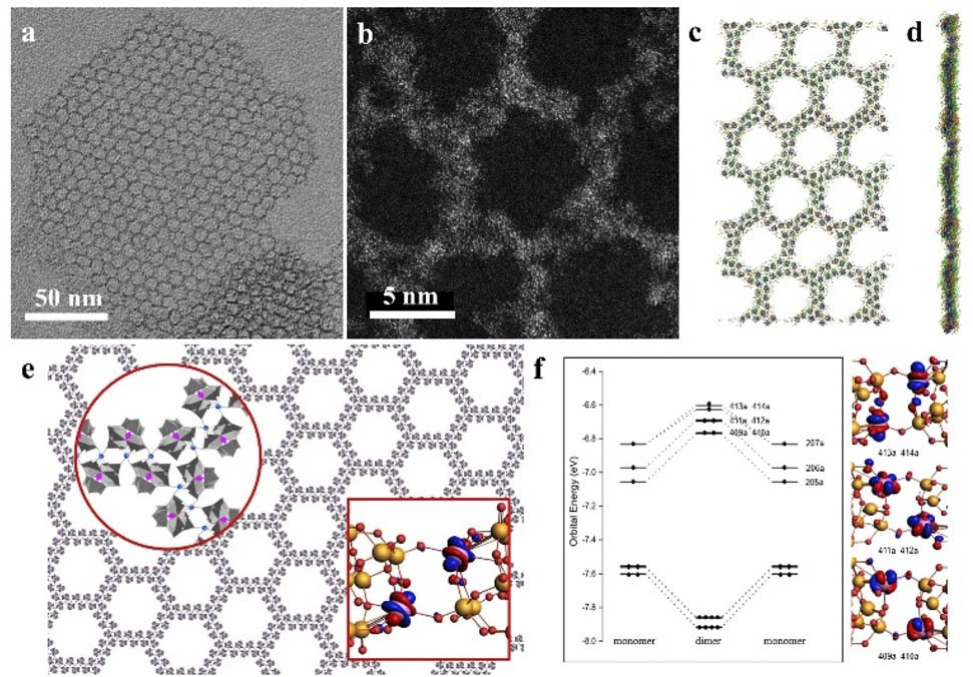
Two-dimensional clusterphenes. (a and b) TEM and HRACTEM images of the monolayer clusterphene. (c and d) MD simulation of the monolayer clusterphene: top view (c) and side view (d). (e) Molecular model of the clusterphene layer. (f) The valence molecular orbital energy-level diagrams and the corresponding frontier Kohn–Sham valence orbitals of POM dimer. Nd, pink; W, gold; O, red; H, white. Reproduced from ref. ^58^.

Recently, our group developed a novel class of 2D graphene-like layered structures using clusters, which is referred to as “clusterphene”.^58^ In this work, a series of lanthanide substituted Keggin type clusters [LnPW_11_O_39_]^4−^ are prepared and employed as cluster building blocks. Monolayer clusterphenes exhibit uniform hexagonal pores, with single-cluster thickness and size up to several micrometers (Fig. 7a and b). In clusterphene layers, POM clusters are directly connected *via* Ln–O coordination bonds, according to the higher coordination number of Ln in clusterphenes than in an individual cluster. The average side length and width of hexagonal pores are 4.5 nm (4 clusters) and 2 nm (2 clusters), respectively, indicating the zig-zag configuration of POM clusters in the 2D framework. MD simulations reveal that the monolayer POM clusters are sandwiched between surface ligands in clusterphene layers (Fig. 7c and d). With the increase in reaction time and ionic strength, the POM assembly will transform from multilayer into monolayer clusterphenes and finally into ultrathin nanobelts. 13 kinds of LnPW_11_ clusters (Ln = Y and Pr–Lu, except Pm) are able to self-assembly into clusterphene construction, and the assembly behavior of the cluster is dependent on the charge-to-radii ratio of the Ln atom.

## 4. Novel properties in cluster assembly

### 4.1. Surface ligand-assisted assembly

In cluster assemblies, clusters are commonly connected in two ways: (1) through interactions between surface ligands or molecular linkers; (2) *via* direct bonding to share atoms in addition to electrons. In the first case, interactions and charge transfer between clusters and surface ligands can significantly influence the overall electronic structure, leading to changes in spectral, electronic and catalytic properties. The nanoribbons (Fig. 1b) assembled from Au_25_(*p*-MBA)_18_^−^ clusters exhibit enhanced luminescence, contributed by the extensive aurophilic interactions.^38^ The nanoribbons could deliver stable and bright red emission with an absolute quantum yield (QY) of 6.2% at room temperature, which is significantly higher than that of the Au_25_(*p*-MBA)_18_^−^ building block (QY ≈ 10^−4^). By introducing Zn^2+^ ions into non-emissive Au_4_(MHA)_4_ clusters, Hyeon *et al.* reported a highly fluorescent gold cluster assembly with a QY of ∼90%.^61^ The unique aurophilic interactions among Au_4_ clusters produced radiative channels, and the coordination of Zn^2+^ with the carboxylate group rigidified the chemical environment and delayed vibrational relaxation, which were all responsible for the ultra-bright greenish-blue fluorescence of gold cluster assembly.

The cluster configuration and inter-cluster distance may also influence the electron transport in cluster assemblies. The electrical transport properties of the fibrous assembly of Au_21_ clusters (Fig. 1d) can be modulated through tailoring of the associated counterions.^39^ The average electrical conductivity with [AgCl_2_]^−^ (1.44 × 10^−8^ S m^−1^) is two orders of magnitude smaller than that of the [Cl]^−^ counterion (2.38 × 10^−6^ S m^−1^), due to the altered configurations of the interacting π–π pairs of surface ligands. In POM single-cluster assemblies (Fig. 5), the average distance between adjacent clusters varies in nanowires (1.27 nm), nanorings (1.25 nm), and 3D superstructures (1.22 nm), due to their different inter-cluster configurations. 3D superstructures show higher sensitivity toward hydrogen peroxide electrochemical sensing compared with nanowires, nanorings and individual clusters, due to the shorter inter-cluster distance and promoted electrical transport in assembled structures.

[P_2_W_18_O_62_]^6−^ assemblies show promoted photochromic properties and photocatalytic activity, due to partial reduction of W^VI^ to W^V^ under visible light. The (CTA)_3_(TBA)_3_P_2_W_18_O_62_ supramolecular gels (Fig. 2f) display good reversible photochromism, which can change into dark blue under 3 hours of irradiation and fade in the dark after 24 hours.^56^ The helical microporous nanorods (Fig. 2e) would become blue after 60 seconds of xenon lamp irradiation, and turn back to white in 30 minutes by the introduction of oxygen.^55^ Moreover, the nanorods show enhanced photocatalytic activity for toluene oxidation, with a conversion rate of 2254 μmol h^−1^ g^−1^, that is 50 times higher than that of the K_6_[P_2_W_18_O_62_] building block. Metal-substituted single cluster assemblies can be regarded as single-atom catalysts, where the metal atoms with a well-defined coordination environment and uniform distribution serves as the active sites. The Mn-based 2D superlattices (Fig. 6a) exhibit enhanced catalytic activity and stability toward olefin epoxidation at room temperature, with TOFs of up to 4-fold that of Mn-porphyrin catalysts, which is due to the highly ordered cluster arrangement and POM supports as an electron buffer.^60^

**Fig. 8:**
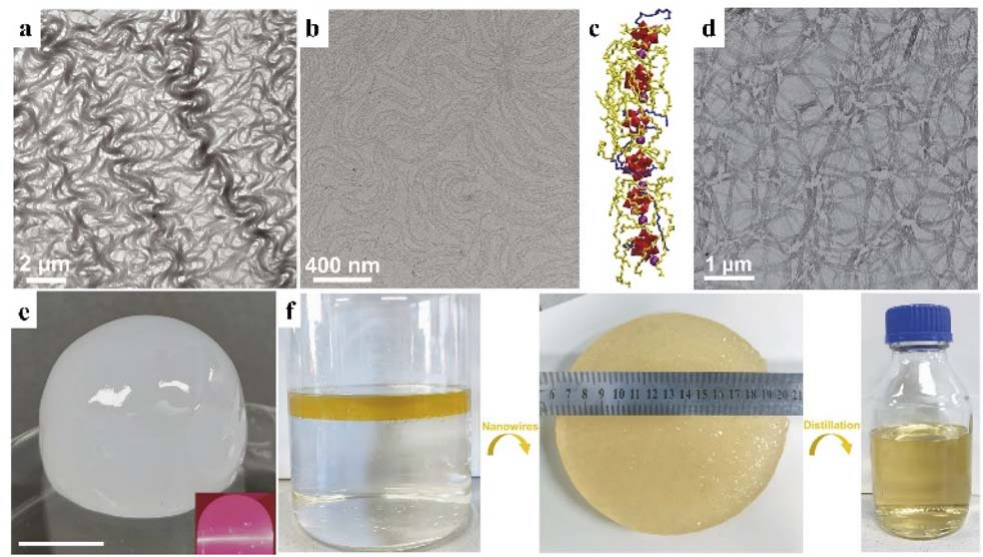
Ca–POM nanowire-based organogel. (a and b) TEM images of nanowires. (c) Structural diagram of the nanowire. Purple: Ca^2+^, red: POM cluster, and yellow and blue chains: oleylamine. (d) TEM image of nanowire networks. (e) Photograph of the nanowire-octane gel. (f) Photographs of the oil spill recovery process with nanowires. Reproduced from ref. 62.

Sub-nanometer nanowires with an ultrahigh aspect ratio can form three-dimensional networks and gels due to their various conformations in dispersions. Therefore, volatile organic molecules with weaker intermolecular forces can be trapped by the use of sub-nanometer nanowires. Recently, our group developed Ca^2+^ bridged [PW_12_O_40_]^3−^ cluster nanowires of 1 nm in width through a facile room-temperature synthesis.^62^ The Ca–POM nanowires exhibited high flexibility, similar to polymer chains (Fig. 8a and b). The nanowires could form a 3D network (Fig. 8d) in organic solvents of octane, cyclohexane and toluene, trapping the volatile organic molecules into organogels with a mass fraction of nanowires as low as 0.53% (Fig. 8e). Ca–POM nanowires can be reused more than 10 times through distillation and centrifugation to remove solvents in gels. Moreover, these cluster-based nanowires can be applied for oil spill recovery, by locking the petrol on water and forming a gel with nanowires (Fig. 8f).

### 4.2. Supermolecular assembly

Due to the high similarity in orbital shapes, chemical properties and electronic behaviors, clusters could be classified as superatoms with a well-defined valence.^63,64^ The valence and atom doping would significantly influence the geometry, electronic energy levels and reactivity of a superatom cluster.^65–67^ The combination of superatoms to share atoms in addition to electrons leads to the formation of a supermolecule.^68,69^ Similar to atomic orbital hybridization, the orbitals of supermolecules could mix and transform into several hybridized superatomic orbitals, resulting in notable changes in electronic structures and properties.

**Fig. 9:**
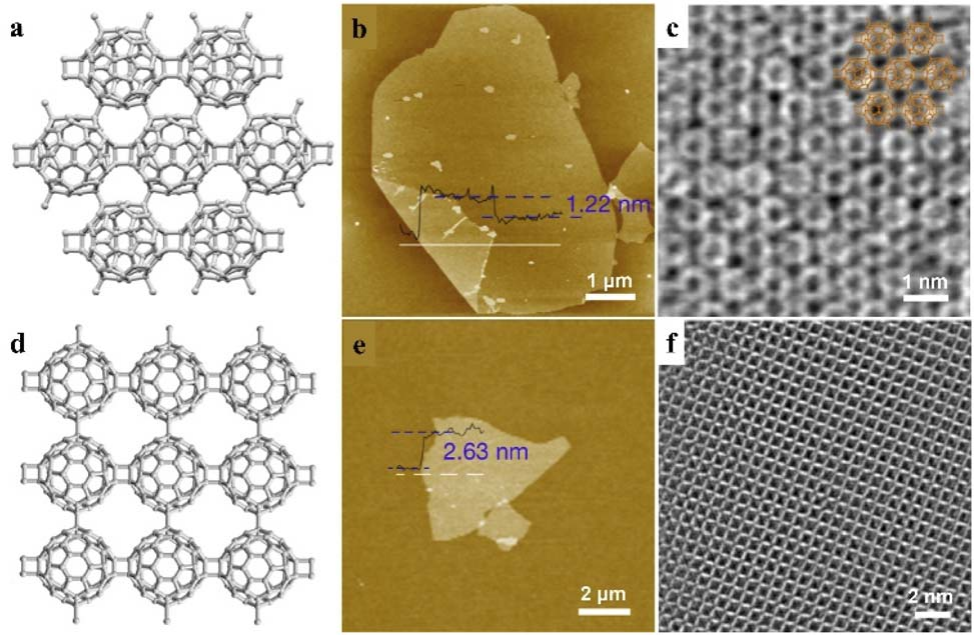
Monolayer fullerene network. (a–d) The framework structures of the bulk single crystal of qHP C_60_ (a) and qTP C_60_ (d). (b–e) AFM images of monolayer qHP C_60_ (b) and few-layer qTP C_60_ (f). (c–f) STEM images of monolayer qHP C_60_ (c) overlaid with chemical structures and few-layer qTP C_60_ (f). Reproduced from ref. ^70^.

Two-dimensional materials show interesting physical and chemical properties due to their unique electronic properties confined in a sub-nanometer layer. Zheng *et al.* reported the covalent bonding of fullerene (C_60_) into a 2D periodic nanocluster network structure (Fig. 9a–c).^70^ By changing the ratio of intercalated Mg, polymeric C_60_ in closely packed quasi-hexagonal (qHP) and quasi-tetragonal (qTP) phases was prepared at atmospheric pressure. With the slicing of tetrabutylammonium cations, monolayer and few-layer polymeric C_60_ can be exfoliated from the quasi-hexagonal and quasi-tetragonal bulk single crystals, respectively (Fig. 9b, c and e, f). Due to the bridge bonds on C_60_ clusters, the π-state carbon atoms in each C_60_ changes into 52 and 54 in qHP and qTP C_60_, which may lead to notable changes in electrical properties. The monolayer qHP C_60_ shows semiconductor properties, with a transport bandgap of ∼1.6 eV, which is distinct from the free C_60_ cluster as an insulator. Moreover, the asymmetric lattice structure of monolayer polymeric C_60_ results in significant in-plane anisotropic properties in phonon modes and conductivity, indicating the unique properties and promising potential of this supermolecular cluster assembly.

The catalytic activity of the POM cluster can be attributed to the Mo/W/V/Nb/Ta atoms in the oxygenated polyhedron framework, and the overall performance derives from the synergetic effect of all metal atoms. During the redox of POM clusters, the additional electrons would delocalize among metal atoms, for which it is the entire cluster that serves as the superatom site for the catalytic redox reaction. Clusterphenes can in turn be compared to a super-molecule, due to the high structural ordering and similar chemical environments of directly bonding POM clusters (Fig. 7e).^58^ The electron delocalization in clusterphene layers efficiently reduces the activation energy for the catalytic redox reaction, which brings about dramatically enhanced catalytic activity and stability towards olefin epoxidation. Monolayer clusterphenes exhibit high yields of 81%–100% for different olefins, and turnover frequencies (TOFs) up to 76.5 times that of cluster building blocks. In clusterphenes, the energy of frontier valence electrons of metal atoms shifts up (Fig. 7f), and the electrons are easier to lose compared with individual clusters, due to the orbital interactions between POM superatoms. Clusterphenes can also act as a new type of support for the construction of single-atom catalysts, where extraordinary properties may emerge from large-scale in-plane electron delocalization. In addition, the electronegative and rich-substitutional surface of clusterphenes may provide various options for the metal active sites with enhanced stability and precisely controlled coordination environments. The unique constructions and properties of clusterphenes may enlighten the design and synthesis of nanomaterials in both fundamental standpoints and applications.

## 5. Conclusions and outlook

The self-assembly of clusters into nanomaterials offers a feasible approach for the functionalization of clusters with tailored properties. In the past few decades, a wide variety of cluster assemblies have been fabricated, driven by the synergetic effect of multiple non-covalent interactions. In this perspective, we summarize the cluster assemblies at the nanometer and sub-nanometer scales, including building blocks of noble metal and POM clusters. Due to the surface properties, noble metal clusters are close to isotropic building blocks from the viewpoint of self-assembly. The oriented assembly of noble metal clusters can be realized by the assistance of specific surface ligands. POM clusters with high symmetry tend to assemble into layered structures, and the dimension of the assembly is dependent on the shape of cation surface ligands. By the application of a two-phase approach, metal-substituted POM clusters are able to connect directionally through metal sites, forming single-cluster assemblies with well-defined structures and controllable morphologies and properties at the molecular level. Interactions between clusters and their superatom orbitals bring about a significant change in their electronic structures, because of which enhanced fluorescent, electronic, and catalytic properties have been observed in these cluster assemblies.

Although sub-nanometric cluster assemblies show intriguing structures and properties, challenges still exist in the continuous step forward. There is a broad space for future research through the use of both theoretical and experimental methods. The rational design of cluster building blocks and molecular linkers (including surface ligands) are the primary issue. The surface modification of noble metal clusters *via* covalent bonds may produce anisotropic entities with specific connective sites, and the surface properties and assembly behavior can be tuned precisely by the ratio and location of surface function groups. Multi-metal substituted POM clusters are also available candidates, with enriched metal sites for potential modification. A greater variety of assemblies can be expected, and the synergetic effect of multi-metal active sites in these assemblies may bring about enhanced catalytic activity compared to their mono-metal counterparts. The combination of clusters and other sub-nanometer building blocks gives another option, such as inorganic nuclei, polyhedral oligomeric silsesquioxanes (POSSs), cucurbiturils, and fullerenes. On the other hand, sub-nanometer cluster assembly offers a well-defined model for the investigation of the relationship between the structure and properties. Exceptional properties may arise from the direct chemical bonding of sub-nanometer building blocks, due to orbital interactions and electron delocalization. The in-depth understanding of the solution behaviors and properties of sub-nanometer clusters may inspire the design of cluster assemblies, from basic points to applications.

## Author contributions

Conceptualization and writing – original draft, Q. L.; conceptualization and writing – review & editing, X. W.

## Conflicts of interest

There are no conflicts to declare.

## Supplementary Material
